# Biotechnological Innovations and Therapeutic Application of Pediococcus and Lactic Acid Bacteria: The Next-Generation Microorganism

**DOI:** 10.3389/fbioe.2021.802031

**Published:** 2022-02-14

**Authors:** Sunday Bulus Peter, Zhina Qiao, Hero Nmeri Godspower, Samaila Boyi Ajeje, Meijuan Xu, Xian Zhang, Taowei Yang, Zhiming Rao

**Affiliations:** The Key Laboratory of Industrial Biotechnology, Ministry of Education, School of Biotechnology, Jiangnan University, Wuxi, China

**Keywords:** probiotics, *Pediococcus*, biotherapeutics, bacteriocin, genome editing, deep neural network

## Abstract

Lactic acid bacteria represent a worthwhile organism within the microbial consortium for the food sector, health, and biotechnological applications. They tend to offer high stability to environmental conditions, with an indicated increase in product yield, alongside their moderate antimicrobial activity. Lack of endotoxins and inclusion bodies, extracellular secretion, and surface display with other unique properties, are all winning attributes of these Gram-positive lactic acid bacteria, of which, *Pediococcus* is progressively becoming an attractive and promising host, as the next-generation probiotic comparable with other well-known model systems. Here, we presented the biotechnological developments in *Pediococcal* bacteriocin expression system, contemporary variegated models of *Pediococcus* and lactic acid bacteria strains as microbial cell factory, most recent applications as possible live delivery vector for use as therapeutics, as well as upsurging challenges and future perspective. With the radical introduction of artificial intelligence and neural network in Synthetic Biology, the microbial usage of lactic acid bacteria as an alternative eco-friendly strain, with safe use properties compared with the already known conventional strains is expected to see an increase in various food and biotechnological applications in years to come as it offers better hope of safety, accuracy, and higher efficiency.

**GRAPHICAL ABSTRACT:**
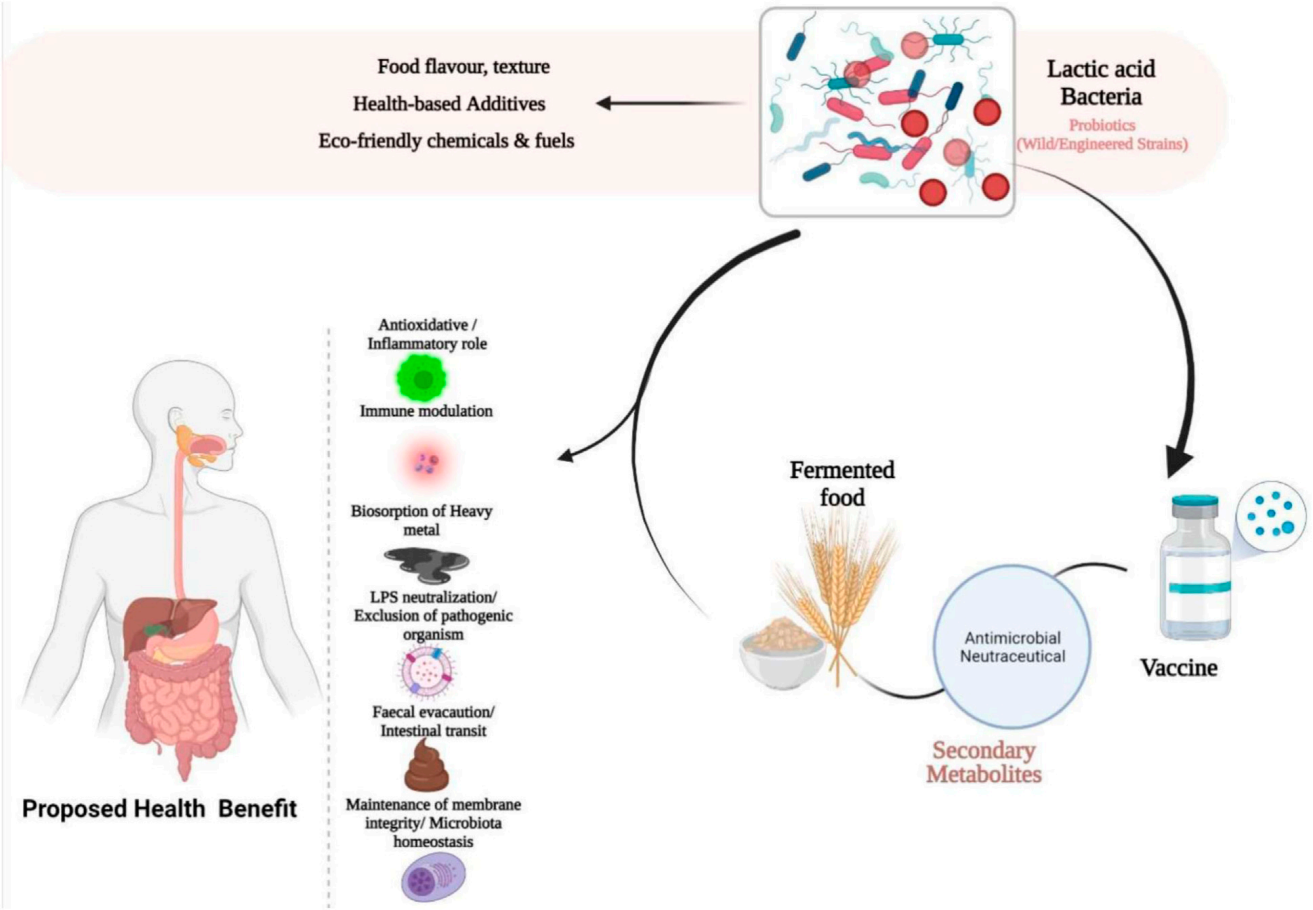


## Introduction

The idea that bacteria are useful can be difficult to understand. Today, however, some are known as friendly, good, or healthy bacteria that are widely used in foods, beverages, and supplements that help promote a healthy digestive tract and immune system. Currently, research continued to support these findings and suggest even more benefits.

Among these bacteria, is lactic acid bacteria (LAB), made up of different sets of Gram-positive, non-pathogenic bacteria that are diverse and taxonomically heterogeneous (George et al., 2018). Generally, they are catalase negative and occur as cocci or rod shaped, although pseudocatalase can be seen in rare cases. The genera *Pediococcus*, *Streptococcus*, and certain species of *Lactobacillus* carry out homolactic fermentation. As observed in *Lactobacillus* species, *Pediococcus* can also create a number of enzymes that releases unique food flavor compounds. Heterolactic fermentation on the other hand is performed by bacteria of the genus *Leuconostoc* and some *Lactobacillus* species (González-Centeno et al., 2017). The *Lactobacillus* and *Pediococcus* genera are found within the family *Lactobacillaceae*, made up of 80 identified species and 15 subspecies that are uniquely different on the basis of the outcome of genotyping. So far, the current classification is based on 16S rRNA (ribosomal RNA) and 23S rRNA sequence comparative analysis, for phylogenetic relationships. This catalogs and sequences reveal that the evolutionary lineage is within the clostridial class for *Pediococcus*, with a low G + C ratio less than 50% compared with other related *Lactobacillus* species, ranging from 84% to 87%. Their cell wall is made up of the amino acid sequence Lys-d-Asp, a peptidoglycan or diaminopimelic acid in some cases (Raccach, 2014). The common sources of these genera are plants, cheese, and processed meats, with industrial relevance as starter culture.

The health-promoting and disease-preventing function of LAB in immune modulation, gut epithelial barrier integrity maintenance, antioxidative capacity, with their important safe record of application in food processing, has warranted the generally recognized as safe (GRAS) status for consumption. In accordance with the Qualified Presumption of Safety (QPS) recommendation, *Lactobacillus*, *Leuconostoc*, *Bifidobacterium*, *Streptococcus*, and *Pediococcus* genera have been considered safe based on taxonomy and appropriate identification at species level (Ricci et al., 2018). In order to properly convey the expected health benefits, the incorporation of beneficial organisms, prebiotics, or symbiotics into food products is important for the gastrointestinal tract (GIT) microbiota and of great technological concern. The right dosage of probiotic, therefore, is required during transit, as it passes through the GIT. In protecting probiotics, enteric coating, microencapsulation, and prebiotic usage are directed toward stimulating the proliferation and activity of the required bacteria, by initiating low-redox potential environment and other factors that address their sensitivity to metabolites formed in the course of growth or as co-adjuvant with other starter culture (Davani-Davari et al., 2019). The generation of off-flavors in food are posed by some metabolites; however, mixed culture with inducer strains, might result in growth-enhancing metabolite production or oxygen content reduction by adjuvant starter culture (Kongo and Malcata, 2016). Withal, the molecular processes attributed to these probiotic bacteria in exerting their health-enhancing benefits is relatively unknown; therefore, precise molecular processes of probiotics action are required.

As the gastrointestinal tract remains the pathway that allows the passage of food and removal of solid waste from the body, it is also understood that the gut microflora that occupies it plays a unique function in various facet of preserving human health, including promoting nutrient digestion and absorption, defending against pathogens, and modulating the immune system. A variety of disorders, like inflammatory bowel disease, asthma, obesity, cardiovascular and autoimmune conditions, are associated with gut microbiota imbalance (Bull and Plummer, 2014). Recently, with the growing understanding of the human microbiome, to prevent and cure some health conditions, probiotics have received considerable attention in bioengineering to support human health. Helped by the extended creation of tools and methods, probiotics are engineered as live biotherapeutics for studying and prevention of many diseases (Mazhar et al., 2020). By stabilizing the gastrointestinal tract, it might offer health-promoting benefits.

Genomic proves for adaptations to the GIT *via* cell-surface anchoring proteins associated with the intestinal barrier causes these bacteria to induce an increase in polysaccharides diversity that are directed toward degradation and the expression of host genes innate immunity. Analogy with completely sequenced LAB genomes shows that the primary impetus of evolution within these genomes is lateral gene transfer. Genomic analysis to a great extent has unravel the core mechanisms by which “Probiogenome” encodes key functions that regulates the growth, survival, signaling, fermentative mechanism, and relatively, the ultimate potential of probiotic activities within multifaceted microbial and host biota (Goh and Klaenhammer, 2015). The evolutionary distance between distant phyla might have an effect on the array of gene availability or its absence in a given set of genomes. Therefore, engineering, in order to maintain a favorable balance in the intestinal microbiota, is essential for a better health status.

Despite LAB therapeutic prowess, these microbes could also be employed to manufacture substances that are currently unavailable, owing to a number of favorable characteristics (Bosma et al., 2017), resulting in the introduction of novel products and microbially produced compounds with the capability of replacing existing products. This might allow for an alternative and cost-effective manufacturing methods in sustainable bulk biochemical production (Bosma et al., 2013; Budzianowski and Postawa, 2016; Budzianowski, 2017), with applications in food, medicine, and pharmaceutical industries. LAB has been utilized in the production of low-calorie polyols including high value products like mannitol and sorbitol produced naturally by several heterofermentative and homofermentative LAB as well (Bozell and Petersen, 2010). Fructose is employed as a carbon source as well as an electron acceptor in certain LAB, where it is transformed to mannitol by mannitol dehydrogenase (Saha and Racine, 2011). Mannitol phosphate dehydrogenase and lactate dehydrogenase (LDH) enzymes were inactivated in *Lactobacillus plantarum* for sorbitol synthesis, and a sorbitol dehydrogenase gene was overexpressed, allowing more fructose 6-phosphate to be converted to sorbitol 6-phosphate (Nissen et al., 2005; Ladero et al., 2007). In addition, there has also been interest in producing xylitol from LAB, a low-calorie sugar alcohol with five carbons that is not formed naturally by LAB. In a *Lactococcus lactis* strain modified with xylose reductase from *Pichia stiptis* and a xylose transporter from *Lactobacillus brevis,* and cultured in a glucose-limited fed-batch cultivation method with high xylose concentrations, xylitol synthesis was observed (Nyyssölä et al., 2005). Lactate may be converted to 1,2-propanediol (1,2-PDO) by *Lactobacillus buchneri*, *Lactobacillus brevis*, and *Lactobacillus fermentum*, a polyol commonly utilized in anti-freeze fluids, polyester resins, non-ionic detergents, coolants, and as an addition in cosmetics, nutritional goods, medicines, and colors (Bennett and San, 2001; Oude Elferink et al., 2001; Bosma et al., 2017).

## Exploiting powerful tools in *Pediococcus* and lactic acid bacteria

### Plasmid and Genome-Based Protein Expression System in Lactic Acid Bacteria

**FIGURE 1:**
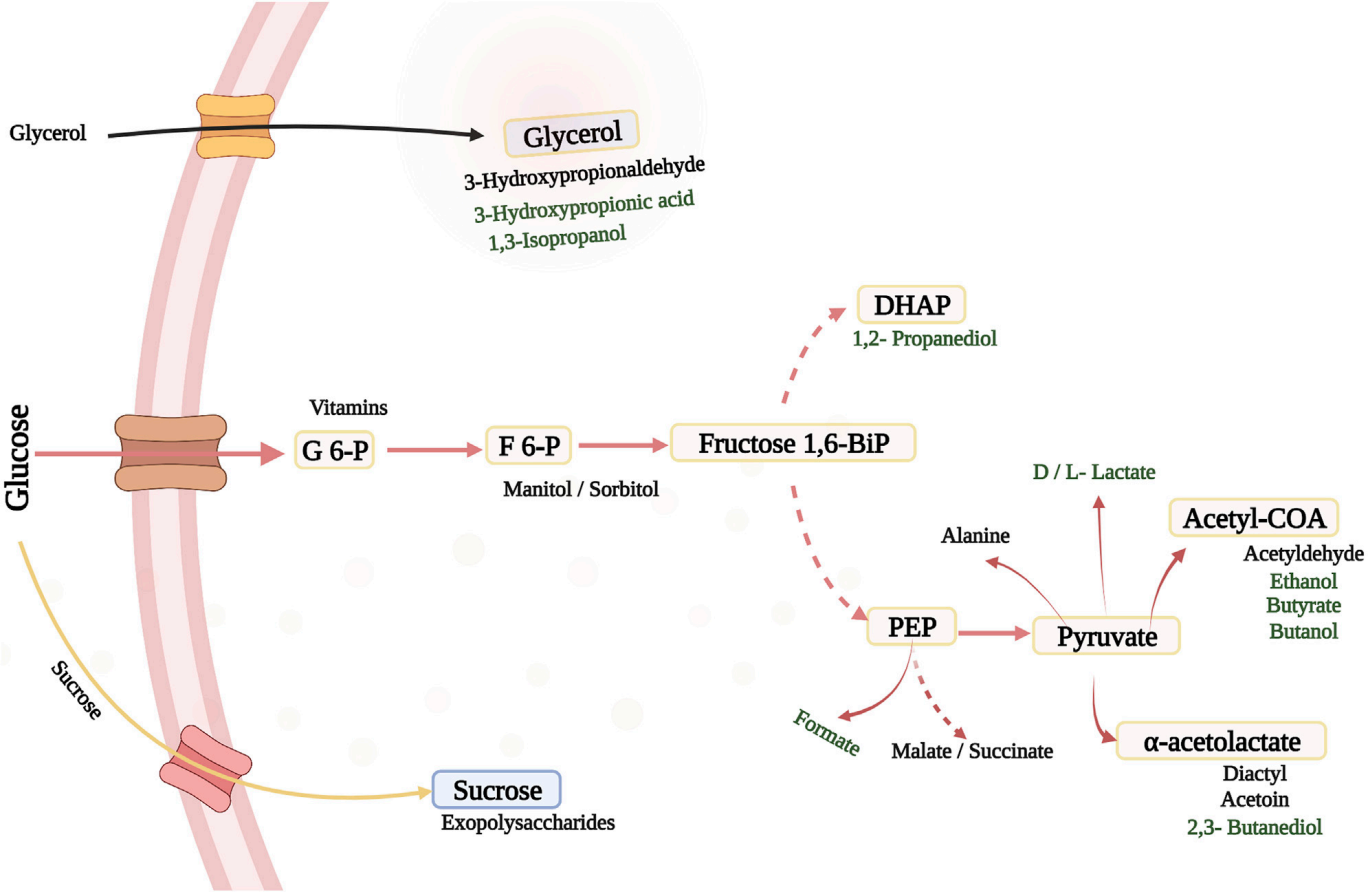
Possible Products in the Natural Metabolic Pathways of LAB and Pediococci under Various Conditions. The dashed arrows indicate different enzymatic steps. Black: taste, texture, medicinal ingredients and health uses; Green: chemicals and fuels. F 6-P, fructose 6-phosphate; DHAP, dihydroxyacetone phosphate; Fructose 1,6BiP, fructose 1,6 bisphosphate; PEP, phosphoenolpyruvate; G 6-P, Glucose-6-Phosphate.

Lactic acid as a globally integrated bio-product, in the manufacture of biodegradable polymers, replacement of oil-based plastics and building-block molecule in the chemical industry (Singhvi et al., 2019), is of great importance, especially in the usage of LAB as new field of application in medicine. New possibilities are posed by genetically modified LAB (Figure 1), which could establish a strong trend in few years.

Plasmids are broadly found in many LAB sources, as a self-replicating material and have gained much attention due to their proximity with a lot of essential functions and industrial importance (O’Connor et al., 2007). *Pediococcus pentosaceus* and *Pediococcus acidilactici* of *Pediococcus species* harbor various plasmids, from 1.82 to 190 kb size that encodes a variety of traits. Some plasmids from *Pediococcus* includes pEOC01, pMD136, pRS4 (A cryptic plasmid), pUCL287 (A cryptic plasmid), and pSMB 74. All plasmids mentioned, with the exception of pRS4, exhibit theta replicating mechanism.

**FIGURE 2:**
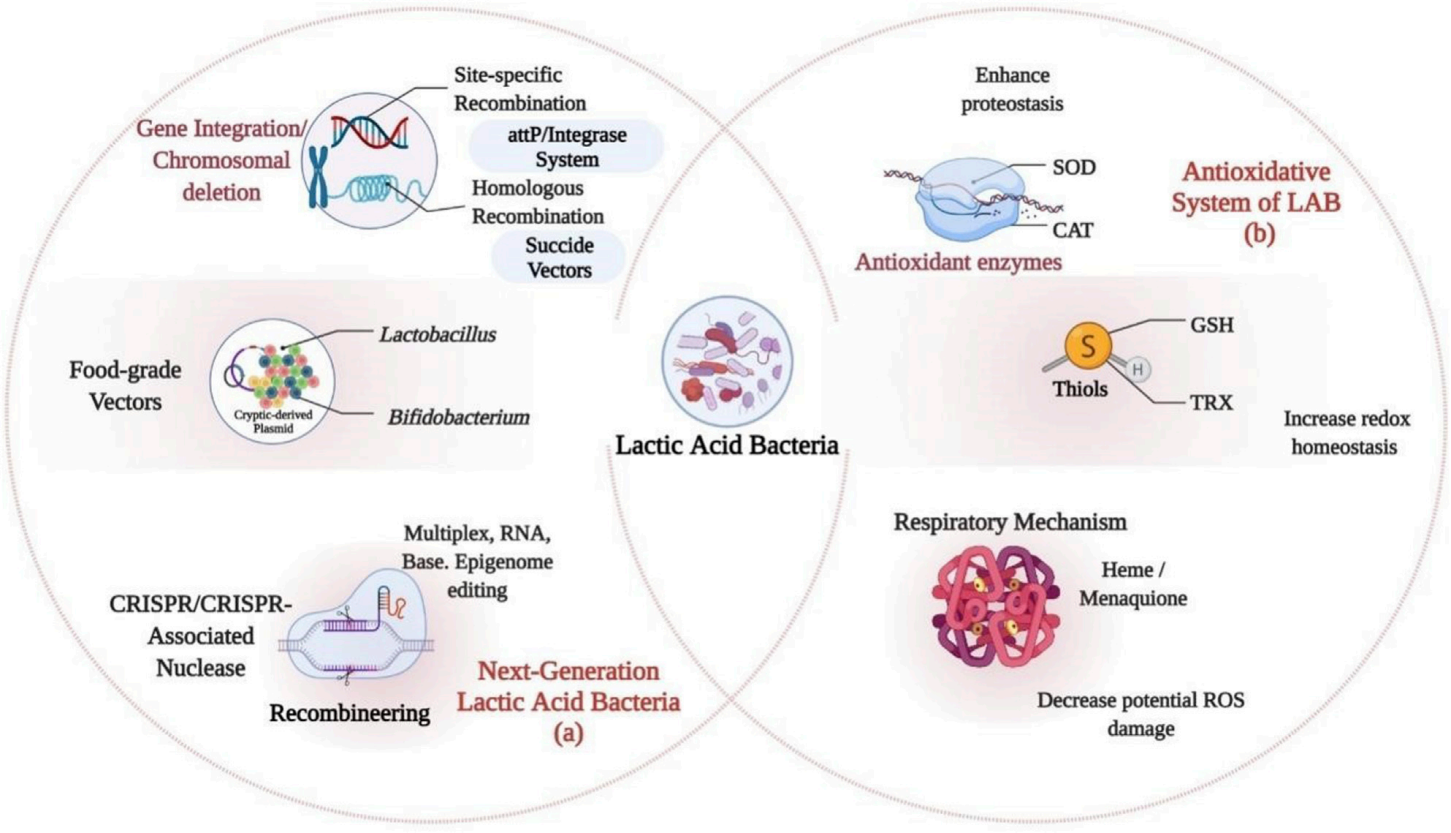
Genomic Approaches and Antioxidative System of Lactic Acid Bacteria (LAB): **(A)** Genome-based approaches to the characterization of promising probiotic strains. **(B)** Antioxidative system of LAB; Thiols, Antioxidant enzymes and Respiratory repertoire which allows them to withstand various environmental stresses that generates highly reactive species or through the NOX (NADH oxidase) like H_2_O_2_. ROS, Reactive oxygen species; SOD, Superoxide dismutase; GSH, Glutathione; TRX, Thioredoxin-thioredoxin reductase system; CAT, catalase.

Plasmids enable direct distribution of multiple copies of recombinant genes and the degree of expression is decided by promoter properties. Inducible promoters are more preferred as they allow efficient control over the expression of recombinant protein than constitutive promoters (Table 1). In the expression of genome-encoded proteins, integration of genome offers lower copy number of gene. However, genome integration brings various support-like stability, acceptability for various species, and void of surrounding effects (Zhu et al., 2015). Introducing classical methods are not often correlated with probiotic effectiveness and safe use. Nevertheless, the use of genome-based approaches for characterization of promising probiotic strains like LAB will help validate the role of these species and might stand as a useful instrument in the regulatory scenario for LAB, allowing them to acquire new attributes and increase in their beneficial characteristics. For human applications, genetic modifications may be used, if antibiotic resistance is eliminated and these bacteria are altered in their genetic code. This could be accomplished by genetic integration or chromosomal deletion, use of food-grade vectors, and clustered regularly interspaced short palindromic repeats (CRISPR) technology (Bravo and Landete, 2017; Castro-López et al., 2021) (Figure 2A).

**TABLE 1:** Plasmid and genome-based protein expression system in LAB.

Organism	Vector expression system	Description	References
Plasmid-encoded System			
***Lactococcus lactis***	P_zn_ zitR and Zirex	*P*_*zn*_ *zitR* built on *P*_*Zn*_ promoter and *zitR* repressor of *zit* operon. Zirex based on pneumococcal repressor *SczA* and *PczcD*. Both requires Zn^2+^ utilization and regulation	(D. and I., 2004; Mu et al., 2013)
	P170	At low pH, the *P170* promoter is upregulated during the transition to stationary phase	Madsen et al. (1999)
	PxylT	Xylose induced; promoter, P*xylT*	Miyoshi et al. (2004)
	SICE	Stress induced	Benbouziane et al. (2013)
	ACE	Agmatine induced; having *AguR* and *P*_*aguB*_ *as* the target promoter	Linares et al. (2015)
	NICE	Nisin induced; based on a two-component signal transduction	Mierau and Kleerebezem, (2005)
Genome-encoded System			
***Lactobacillus jonsonii***	pSA3-based suicide vector (pTRK327)	A non-repetitive vector with sequences homologous to the insertion site	Walker and Klaenhammer, (1994)
***Lactobacillus gasseri***	pTNI with thermosensitive replicon	Site-specific replacement of chromosomal DNA sequence	Neu and Henrich, (2003)
***Lactococcus lactis***	pSEUDO vector	Stable genetic insertion in a pre-set transcriptionally inert part of genome	Pinto et al. (2011)
**Application: Use for over-expression of genes required for functional evaluation, metabolic engineering, membrane proteins expression, secretion of protein and cell surface anchoring. ACE, agmatine-controlled expression system; SICE, stress-inducible controlled expression system; NICE, nisin-controlled gene expression system.**			

These will provide a broad range of opportunities for commercially important *Lactobacillus* and *Bifidobacteria* to be engineered by genetically modifying and improving their therapeutic potential for vaccination or host immune response modulation. Gene editing tools such as transcription activator-like effector nucleases (TALEN), zinc-finger nucleases (ZFNs), CRISPR, and various approaches to omics, and systems biology could allow better comprehension and restructuring of metabolic pathways. These methods will strengthen the awareness of researchers toward the gut microbiome and provide novel routes for probiotic bacteria to be studied (Yadav et al., 2018). CRISPR, as a revolutionary genome editing technology has provided a key bioengineering tool for investigating different diseases with great sensitivity, speed, precision, and a comprehension of biological processes (Yadav et al., 2021). However, an extensive strategy to diversifying the genome engineering toolbox is to discover new CRISPR systems. Recently, developments in detection of specific CRISPR systems and the restructuring of the Cas9 protein for extended functionalities have been made possible. This could improve and expand the efficiency of the Cas9 nuclease.

### Recombineering and CRISPR–Cas System in Lactic Acid Bacteria

Novel techniques have been introduced recently that seems to overcome the time-consuming and relatively inefficient procedures of insertion by classical chromosomal integration techniques. Recombineering allows precision genome engineering, while CRISPR Cas-based engineering helps in putrefying selection for both recombineering and homologous recombination (Table 2; Figure 2) (Doudna and Charpentier, 2014; Ortiz-Velez and Britton, 2017), which help circumvent the need for mutation screening.

**TABLE 2:** New genome engineering possibility with recombineering and CRISPR–Cas system in LAB.

Organism	Vector/system	Characteristics	References
*Lactococcus lactis*	pLABTarget	For introducing sgRNA sequence to target specific genetic loci	Simon et al. (2021)
*Lactococcus lactis*	pNZCRISPR (a single plasmid inducible CRISPR-Cas 9 system)	To drive Cas9 expression and transcription of two nisin promoters, respectively	Berlec et al. (2018)
*Lactobacillus reuteri*	CRISPR-Cas system + (ss)DNA recombineering (RecT-assisted CRISPR–Cas9 system)	Enhances performance in bacteria with low recombineering efficiency; Cas 9 directed toward eliminating unmodified bacterial strains (codon saturation mutagenesis and gene deletions) (100% efficiency)	Oh and van Pijkeren, (2014)
*Lactobacillus casei*	pLCNICK A CRISPR–Cas9^D10A^-based plasmid	Replaces wild-type Cas 9 with Cas 9^N10A^ (nickase), increasing efficiency (25%–65%)	Song et al. (2017)
*Lactococcus lactis* NZ9000	a RecT-assisted CRISPR–Cas9 approach	Easy insertion or deletion of genomic DNA within less time	Guo et al. (2019)
*Lactobacillus plantarum* WJL	CRISPR-Cas system + ssDNA recombineering	A plasmid-encoded template, oligonucleotide donor and an inducible DNA recombinase	Leenay et al. (2019)
*Lactococcus lactis* MG1363	*Streptococcus* pyogenes CRISPR-Cas9	Genome modification of *L. lactis* virulent phage P2 with exact mutations without heterologous recombinases	Lemay et al. (2017)
*Lactobacillus plantarum*	CRISPR interference (CRISPRi) systems	CRISPRi with catalytically dormant form of Cas9 (dCas9) for gene expression. Marker free	(Berlec et al., 2018; Storaker et al., 2021)
*Lactobacillus reuteri and Lactococcus lactis,*	Single-strand (ss) DNA recombineering	Mediates chromosomal incorporation of mutation. Antibiotic selection free (0.4%–16% efficiency)	(Yu et al., 2003; van Pijkeren and Britton, 2012)
*Lactobacillus plantarum* WCFS1	λ-Red-like recombinase system, a (Ds) DNA recombineering	A site-specific recombinase system for precise deletion and replacement with high efficiency	Yang et al. (2015a)
*Lactobacillus casei* BL23	λ-Red-like recombinase system, a (Ds) DNA recombineering	For deletion and insertion of Gfp gene. marker free	Xin et al. (2017)

In LAB and starter cultures of industrial concern, this technology grasps a better assurance for application. Its DNA-encoded, RNA-mediated, and DNA-targeting protective roles offer sequence-specific targeting of DNA (Rath et al., 2015), by producing alteration at the excision site using different pathways, that results in the initiation of double-stranded DNA breaks. These technologies will continue to remodel total bacterial exploitation and the editing of eukaryotic genomes. In CRISPR-based technologies for probiotic organisms. Application of (ss)DNA recombineering merged with CRISPR-Cas selection for gene editing of bacteria has an immense value in the production of industrial bacteria of the next century (Barrangou and van Pijkeren, 2016). CRISPR–Cas systems are extensively found in most LAB with eight subtypes and can be present in bacterial genomes alone or in multiple occurrences. These helps enhance the efficacy of fermentation, allows phage resistance or provide basic perception into starter cultures. Problems, particularly discharge and the release of antibiotic resistance markers, can arise from their engineering. Nevertheless, this may be alleviated by biocontainment of genetically modified LAB in a closed system, use of other selection markers and homologous DNA (Plavec and Berlec, 2020). However, unlike (Ds)DNA recombineering, in which the selection of positive mutants is marker dependent, which allows excision of the selection marker, leaving a scar on the loxP site of the modification locus, in (ss)DNA recombineering, it is uniformly avoided.

Cell metabolism rewiring occurs progressively and often at low throughput, but for efficient microbial cell factories, a combinatorial metabolic engineering method based on an orthogonal trifunctional CRISPR system (CRISPRAID) with three CRISPR proteins, a nuclease-deficient CRISPR protein fused to an activation domain (CRISPRa), nuclease-deficient mutant fused to a repression domain (CRISPRi), and finally a catalytically active CRISPR protein (CRISPRd) have been proposed for an effective metabolic and regulatory network with high performance permitting the examination of all possible gRNA combinations to build optimal cell factories (Lian et al., 2017). This has contributed to a 2.5-fold increase in β-carotene and an improved presence of endoglucanase on yeast surface. The ideal genome editing method for achieving any desired modification with minimal adverse effects is still a work in progress. Nevertheless, unlike previous methods, prime editing has implemented exogenous reverse transcriptase action to “write” DNA modifications directly to genomic DNA using a programmable RNA nickase linked to a reverse transcriptase and prime editing guide RNA (Anzalone et al., 2019). Both the genomic target and the sequence may be edited using approaches. By creating a powerful, inducible, broad-spectrum vector-based mutagenesis system that amplifies mutations above baseline and exceeds the mutation efficiencies and range of commonly used *in vivo* and *in vitro* approach, a mechanism-driven approach could have advantages over current methods as it offers improved random mutagenesis in cells with control, genomic stability, improved efficiency, and wider mutation spectra. The introduction of a system for the detection of antibiotic resistance that overcome chemical mutagens, ultraviolet light, and the mutant strain XL1Red subjected to the same conditions in *E. coli* could allow for the progressive evolution of T7 RNA polymerase variants with the potential to initiate transcription using the T3 promoter on both bacterial and bacteriophage-mediated laboratory evolution procedures (Badran and Liu, 2015).

## Antimicrobial peptides of *Pediococcus*

Biopreservation systems have gained growing attention in recent years, causing consumers to be more conscious of the danger to health triggered by chemical preservatives in foods. Interestingly, most producers of bacteriocin are part of LAB, a category found in food with a historic record of safe utilization in the dairy industry. Since bacteriocins, either refined or released by its producing strains show no health risk issues, they offer an alternative to the use of deleterious preservatives (Silva et al., 2018). The possibility of engineering and improving their biological activity could be realized when the alteration systems in bacterial cell use in processing these proteins are known. In LAB, this is a current frontier and one with a lot of possible rewards.

A proposed universal scheme for bacteriocin alongside the original scheme of classification for lactic acid bacteria bacteriocins by Klaenhammer has been put forward, by integrating the revised scheme of Cotter and establishing linear, globular, and multi-component into the lantibiotics subgroups. Antimicrobial peptides (AMPs) with its broad multifunctional tools to fight microbial infections and modulate immune response of the host enhance or restrict the entrance of cells and harmful chemicals to the infection site, henceforth, protecting cells from serious tissue damage. *Pediococcus pentosaceus* zy-B, produced by bacteriocin PE-ZYB1, shows heat resistance and a wide variety of antimicrobial action with increased electrical extracellular conductivity and bactericidal degradation of *L. monocytogenes* cell membrane integrity (Heng and Tagg 2006; Zhang Y. et al., 2020). For pediocin usage as food additive, the most desired feature is its stability in heterogenous food system. Various bacteriocins from *Pediococcus* contain residues of methionine, the sulfur particle of which might be oxidized, leading to bacteriocin instability. With an emphasis on Pediocin PA-1 methionine residue, substituted Met13 residue with *Ala*, *Leu*, or *Ile* has proven to shield peptide from the effect of oxidation with moderate influence on the action of antimicrobials, while the replacement of *Asp* results in obvious fall in efficiency against indicator strains. Therefore, a relevant point in transforming Pediocin PA-1 into a valuable food additive is to increase its stability by substituting methionine with a different hydrophobic residue to conserve its function (Brumfitt et al., 2002). Continuous research can bring about pediocins with enhanced stability and improved functions. However, acetic acid, which is indirectly associated with the cell center metabolism, influencing and controlling bacteriocin production, in its undissociated state was found to freely disperse and dissociate through the hydrophobic layer of the membrane (Ge et al., 2019), influencing the development, yield, and activity of bacteriocin production.

### Cloning and Expression of Bacteriocin in *Pediococcus*

Pediocins (Bacteriocin Class II) are made by *Pediococcus acidilactic* and *Pediococcus pentosaceus s*trains with GRAS status in certain food applications, characterized by exceptional antimicrobial potency, good thermostability, wide pH range, and efficacy against spoilage and pathogenic organisms. In targeting members of the same species, bacteriocins can exhibit a narrow range of activity; meanwhile, others can show a wider activity for targeting other species and genus. Variants of bacteriocins have proven more significant activity than their native counterpart. This is made possible through DNA shuffling techniques by creating chimeric gene sequences having desirable traits. Bacteriocin like Pediocin PA-1 applies its role by disrupting the cytoplasmic membrane, causing outflow of ions and small molecules (Villalobos-Delgado et al., 2019). Nisin was, until recently, the only bacteriocin to be sold as a food biopreservative. However, a bacteriocin produced by *Pediococcus* species has also been commercially sold under the name Alta 2341^™^ with an effectiveness relatively as that of Nisin against *L. monocytogenes*, *Staphylococcus aureus*, *Pseudomonas*, and *E. coli* (Deegan et al., 2006). Pediocin A, Pediocin N5p, Pediocin L, Pediocin S, Pediocin ACCEL, Pediocin ST18, Pediocin SM-1, Pediocin pK23-2, Pediocin 05–10, Bacteriocin ST44AM, and Pediocin P are among the examples of pediocins (Papagianni and Anastasiadou, 2009). Single or co-adjuvant cultures of lactic acid bacteria producing bacteriocin help regulate adventitious flora and induce cell lysis, which improves food quality and sensory attributes. Recently, the incorporation into bioactive films and coatings of bacteriocins into food surfaces and packaging of bacteriocin-producing LAB has been made possible (Silva et al., 2018). Research findings on the efficacy of bacteriocins as bio-preservatives is remarkable, but the industry is a bit reluctant to commit itself financially to the production of commercial preparations of bacteriocins due to low production rates, costly downstream processing, and the legal problems that could occur from their production.

### Strategies for Production of Other Metabolites from *Pediococcus*

Effect of temperature, acid, osmotic pressure, and oxidative effects are some of the environmental conditions encountered by LAB in the gastrointestinal tract and during food processing that possess a significant effect on their biological functionalities. However, LAB is empowered with adaptation mechanisms that protectively saves them from harsh environmental stresses and gene alteration (Figure 2B) (Aakko et al., 2014). Some LAB like *Lactobacillus fermentum* (KGPMF28, KGPMF2) can survive at 45°C for 24 h. At high temperatures, cellular activities are disrupted with increase membrane fluidity due to degradation and loss of function of proteins and nucleic acids. Condition optimization for γ-aminobutyric acid (GABA) production by *P. pentosaceus* MN12 has provided the basis for the formulation of a GABA-rich fermented product. The use of heat shock proteins and enzymes has recently been applied to reduce biomolecule denaturation and degradation (Chen et al., 2017; Thuy et al., 2021). Understanding LAB adaptation mechanisms at extreme temperatures is crucial for sorting out LAB species as starter cultures and probiotics.

In single-strain fermentation, the development of most products is typically poor. However, improving bacterial cocultures suited for high yield, diversification, and sustainability for fermented products might be required. Fermentation of products made from selected species has shown increased productivity, reduced volatile hexanal off-flavor-generating compounds, and created several attractive flavor compounds (Sharma et al., 2020). In assessing the complex growth of *P. pentosaceus* and *S. cerevisiae* during fermentation, the forms and contents of esters showed an increase in co-culture with *P. pentosaceus*. Also, the concentration of bacteriocins yield by P. pentosaceus 147 increased from 1.92 × 10^4^ AU/ml to 5.12 × 10^4^ AU/ml as coadjuvant with *Lactobacillus plantarum* LE27 (Gong and Qi, 2020). Interestingly, in our laboratory, *C. glutamicum* G01 in coculture with a multimutagenized *L. plantarum* GB01-21 yielded a high concentration of 80.5 g/L, 2.68 g/L/h productivity of γ-aminobutyric acid (GABA) from glutamic acid using glucose from cassava via a two-stage fermentation strategy (Yang T. et al., 2015). These present exceptional strains required for microbial co-adjuvant in increasing desired product yield from a glucose-based substrate for health and food.

Lactic acid bacteria can form protective biofilms that allows its survival in harsh environment. Nevertheless, surrounding factors, like additives, can serve a major function in biofilm formation (BF), pH and addition of sugar supplements, with effect on LAB metabolism, growth, lactate output, and bacteriocin production. Research using *P. pentosaceus* ATCC 43200 grown at pH 5.0–6.0 in MRS medium (control) or in the same amounts after inclusion of 0.5, 1.0, and 1.5% (w/w) of sucrose and inulin. A variation in these parameters existed at pH 6.0 between the control medium and the supplemented media. At exponential level, both sucrose and inulin accelerated *P. pentosaceus* growth (de Souza de Azevedo et al., 2017). Recently reported as a potential threonine producer, *P. pentosaceus* TL-3 showed a boost in threonine output via optimized medium (Lim et al., 2019). These can help ensure a more economical growth medium for prospective large-scale application. However, the addition of certain substances (e.g., honey phenolic extract) might decrease LAB biofilm formation.

## Therapeutic application of probiotic lactic acid bacteria

Undoubtedly, probiotics is currently becoming a great deal of focus in scientific probing and application in prevention or treatment of health conditions (Tables 3 and 4). In the pathology and development of chronic inflammation of digestive tract, the gastrointestinal microbiota is considered a critical factor, through the preservation of gut membrane integrity and regulation of the host immune system. Reduced colitis, decreased weight loss and disease activity index, and short-chain fatty acid formation are but a few unique observed features (Bian et al., 2020).

**TABLE 3:** Application of *Pediococcus* in health-related conditions.

Disorder/disease	Organism/strain	Characteristics	References
Oxidative stress	*P. pentosaceus* ZJUAF-4	ZJUAF-4 administration improved Nrf2 expression and its downstream genes, preserved activity of the intestinal. Exerts antioxidant potential	Hao et al. (2021)
Colitis	*P. pentosaceus* LI05	Incredibly preserved intestinal membrane integrity, modulates the immunological profiles, gut microbiota, and metabolite content	Bian et al. (2020)
Biosorption of heavy metal	*P. pentosaceus* FB145 *and* FB181*, P. acidilactici* BT36	Good human gastrointestinal system tolerance properties, reduced Cd bioaccessibility, and protection against Cr toxicity	(Le and Yang, 2019; Feng et al., 2020)
Shields brush border membrane effect	*P. pentosaceus* GS4	Improved fecal evacuation of cadmium with a reduced tissue deposition effect, decreased hyperplasia, reduced invasion of lymphocytes, and enhancement of BBM-based disaccharidases	Dubey et al. (2019)
Aging	*P. pentosaceus* DK1	Improved collagen in UVB-irradiated human skin fibroblasts	(Ji Hoon et al., 2019)
Melanogenesis	*P. acidilactici* PMC48	Reduced overproduction and accumulation of melanin that induces skin darkening and abnormalities	(Sukyung et al., 2020)
Intestinal inflammation	*P. pentosaceus* AK-23*,* ON89A*, P. parvulus* 2.6	*P. pentosaceus* AK-23; ability to bind LPS, neutralizes LPS	Asami et al. (2017)
		*P. Parvulus* 2.6 excretes a ropy EPS with probiotic potential	

**TABLE 4:** Summaries of available therapeutics from various recombinant LAB.

Strains	Therapeutic products	Health-related condition	References
*Lactococcus lactis*	Interleukin-10, proinsulin	Diabetes mellitus (Type I)	Steidler et al. (2000)
*Lactococcus lactis* NZ9000	Interleukin-12	Asthma	Bermúdez-Humarán et al. (2003)
*Lactococcus lactis* NZ9000	HSP65–6P277	Diabetes mellitus (Type I)	Ma et al. (2014)
*Lactococcus lactis* NZ9000	Kisspeptin	Colorectal cancer	Zhang et al. (2016)
*Lactococcus lactis* NZ9000, *Lactobacillus casei*	HPV-16-E7	Human papillomavirus 16-induced cancers	(Bermúdez-Humarán et al., 2005)––
*Lactococcus lactis* IL1403	Interleukin-6	Adjuvant	Li et al. (2015)
*Lactococcus lactis* CHW9	Peanut allergen Ara2	Hypersensitivity intolerance (Type I)	Glenting et al. (2007)
*Lactococcus lactis* NZ9800	Birch allergen Betv1	Hypersensitivity intolerance (Type I)	Daniel et al. (2006)
*Lactococcus lactis* MG1363	Glycosylated tyrosinase-related protein-2	Skin cancer	Kalyanasundram et al. (2015)
*Lactobacillus plantarum* NCL21	Japanese cedar pollen allergen Cry j1	Hypersensitivity intolerance (Type I)	Ohkouchi et al. (2012)
*Lactobacillus pentosus* SS6	γ-Amino butyric acid	Anxiety, hypertension	Zhong et al. (2019)
*Lactobacillus acidophilus* PTCC1643	Hyaluronic acid	Dermatitis, wound healing	Chahuki et al. (2019)

The resultant effect of exposure to highly reactive intermediates is oxidative damage to proteins, lipids, and nucleic acids. This is due to instability in the production of these intermediates and the antioxidant activity of the organism (Tan et al., 2018). Interestingly, the amount of oxidative damage increase and NAD^+^ decline has been linked to aging symptoms and can be at the root of a number of age-associated diseases. According to recent proof, increasing NAD^+^ levels might help reduce or even reverse the effects of aging, as well as retard the progression of age-related diseases. At the moment, a great deal of research has been centered on foodstuff sources, nutritional ingredients, and components that confers antioxidant result in humans and other life forms (Amaretti et al., 2013). However, the principal mechanism by which LAB relieves oxidative stress and intestinal damage is not fully comprehended. Another increasing health-related concern is the enormous increase level in heavy metal toxicants in human body. *P. pentosaceus* (FB145, FB181) reduced cadmium bioaccessibility by 46%, serving as an effective biosorbent to prevent cadmium harmful effect and reduce its assimilation into the human body (Le and Yang, 2019), thereby reducing toxicity to essential organs.

The capacity to degrade histamine, reduce bile salts from cholesterol, strain susceptibility to gastrointestinal diseases, adhesion to CaCO_3_ cells, antibiotic tolerance, and the removal of virulence genes are of importance as a unique attribute of a good probiotic strain. *Lactobacillus paracasei* L3C21M6 offers an increase in cholesterol and histamine-lowering potentials, absence of virulence genes, and good susceptibility to relevant antibiotics (Amaral et al., 2017; Domingos-Lopes et al., 2020). *Pediococcus parvulus* shows no ability to produce tyramine, histamine, or putrescine (Garai et al., 2007), suggesting that this ability may be strain specific instead of being associated to certain bacteria species. The main cause of sepsis is by lipopolysaccharide (LPS), which is linked to an increased death toll in patients. There are currently no effective therapeutic agents available to prevent patients from sepsis, which is depicted by LPS-mediated tissue damage and organ breakdown (Hu et al., 2013). Among intestinal bacteria, *Escherichia coli* or *Salmonella* spp. possesses lipopolysaccharide, which could cause inflammation of the intestines of humans. LPS, particularly its lipid A portion, is actually toxic. Using *Pediococcus pentosaceus* AK-23, with the capacity to bind LPS (Asami et al., 2017; Kawahara, 2021), these harmful bacteria might be managed effectively. Additionally, melanocytes, a specialized pigment-generating cell, houses pigmented particles called melanosomes, responsible for the production of melanin. Previously, a melanin synthase of melanocytes that can suppress tyrosinase, from kimchi-derived *Pediococcus acidilactici* PMC48, was developed and used as a medicinal component to actively fight the overproduction and accumulation of melanin that induces skin darkening and abnormalities (Sukyung et al., 2020). In anticipation, this could be of great importance as a basic material for melanin degradation in pharmaceutical products.

## Challenges and future outlooks

Probiotic candidates like *Pediococcus* and other well-known LAB are chosen to assess their sufficient tolerance to physiological barriers. Further research is required, however, to explain the awareness of their metabolic capacities, main adaptation characteristics, health and physiological functions. Bioengineering techniques can create characteristics that may be very rewarding, yet, they can also be detrimental, if not properly handled. Nevertheless, as the consumption pattern of minimally processed and preserved foods is growing, the use of pediocins of the food industry, as moderate antimicrobials, may provide solutions and alternatives to traditional means of preservation. Pediocin is projected to see more uses in medicinal application in the future (Bron and Kleerebezem, 2011). Understanding the mechanisms in *Pediococcus* and certainly other probiotic species will result to the creation of a molecular toolbox for applications in various sectors.

### Nanotechnology and Microencapsulation

Probiotics are primarily delivered to humans by dairy-based products. The next likely food category appearance of this bacteria would be in non-dairy products. Methods that will provide probiotics with the requisite safety allow them to step beyond medicinal and supplemental uses into the field of food ingredients. Probiotics can be protected from environmental stresses using microcapsules and released in a regulated manner. Its absorption in the intestinal tract can also be aided by mucoadhesive polymers (Razavi et al., 2021). Nanocomposite, nano-emulsification, and nano-structuration will help open up a whole new chapter of possibilities for probiotics applications. Food with nanostructures might possess the ability to enhance flavor, texture, consistency, and increase yield in other important metabolite production. By using alginate, these can be applicable in several probiotic strains, with high survival rate besides non-encapsulated cells at low pH and moderate heat treatment. Microencapsulation techniques with gelatin, to offer a protective coat to acid-sensitive *Lactobacillus* and *Bifidobacterium*, have also been tried. Encapsulation of food additives has the potential to prolong product shelf life, enhance interaction with specific receptors (Sekhon, 2010), and function as *de novo* vaccines (Ravi Sankar and Dastagiri Reddy, 2010) to modulate immune responses.

### Bioinformatics and Biosensors

Current approaches in “Probiogenomics” study have incited much interests and provided means for studying probiotic mechanisms and the identification of new strains from broad microbial sources. PROBIO and other probiotics database were put in place for these efforts. With the continuous change in sequencing and bioinformatic techniques, genomic sequence data for the species of interest could allow for improved regulation, optimization, and recognition of novel genes necessary for survival during processing and technical versatility of probiotics by allowing gene expression profiles of the strain during fermenter growth and different production steps (Ahmed, 2003; Song et al., 2021). In the future, this might help to tailor the technical properties of probiotic strains. Also, detecting quorum-sensing signal of pathogenic bacteria in GIT by release of reporter enzymes (Mao et al., 2018), generation and secretion of anti-enterococcal peptides upon sensing *Enterococcus* peptide pheromone with precise suppression of *enterococcal* growth (Borrero et al., 2015), the use of nisin-producing strains and immunity proteins in combination, to express reporter proteins in a tractable spatial manner (Kong et al., 2017), and an independent inducible degradation of marked proteins with accurate control of the abundance of specific proteins (Cameron and Collins, 2014), all these ushers probiotics and LAB to the world of synthetic engineering for therapeutic applications.

### Artificial Intelligence, Neural Network, and “SynBio” Approaches

The anti-malarial medicine artemisinin took a very long time to develop; however, we could revolutionize what we can achieve with bioengineering if we can grow new cells to specification in a matter of weeks or months rather than years. Scientists are now working on a new tool that will adapt machine learning algorithms to the demands of synthetic biology in order to methodically steer progress and avoid a lengthy process (Zhang J. et al., 2020; Radivojević et al., 2020). The options are limitless. Scientists will no longer have to spend years learning about each part of a cell and what it does in order to manipulate it; instead, using a small set of training data, the algorithms will be able to predict how changes in the DNA of a cell or biochemistry will affect its behavior, then make recommendations for the next engineering phase, as well as probabilistic predictions for achieving the desired goal.

One of such development is Unified Representation (UniRep), a versatile summary of fundamental protein features that applies deep learning to unlabeled amino acid sequences, in order to distill the essential properties of a protein into a statistical representation. This tends to integrate unpredictable protein sequences into fixed-length vectors that resemble the capabilities, function, and configuration of critical proteins in the absence of structural or evolutionary histories. UniRep pushes protein computing to move straight from sequence to design by learning a novel base from scratch (Alley et al., 2019). Surprisingly, the UniRep might be used to rapidly generalize distant and invisible sections, revamp protein engineering roadmaps, or, at best, facilitate the detection of sequence variants that are not accessible to conventional procedures. Although it is limited by sampling bias in the length of the training sequence data, the size and coverage of the sequence databases and specific deep learning computer hardware has improved exponentially. UniRep-guided protein design pledges to boost the rate of manufacturing biosensors, DNA and protein binders, and develop genome-editing enzymes. Experts have also sought for decades to use handcoded heuristic algorithms to prescribe the rules of chemistry to computers. Discovering retrosynthetic routes using Monte Carlo tree search and symbolic artificial intelligence (AI) might assist in delivering high-quality findings. This scheme uses an expansion rule network to help choose the most efficient retrosynthetic steps more quickly than the usual computational search approach. Almost every published answer in organic chemistry has been trained into these deep neural networks (Segler et al., 2018). This makes the target molecules simpler precursors that facilitates the development of a suitable strategy for the synthesis of small organic compounds. This could be crucial in keeping the machine a useful chemical synthesis assistant capable of solving the most pressing problems in agriculture, health, and materials science.

### Phage-Assisted Continuous Evolution

Soluble expression phage-assisted continuous evolution (SE PACE) is a scheme for swiftly developing proteins with high solubility in expression, applicable in many proteins to increase their soluble expression through the rapid generation of improved variants without library cloning, or to improve time-consuming methods that allows protein-driven evolutionary methods. By using a PACE compatible splitintein pIII that functions as an AND gate to join two orthogonal positive selections, SE-PACE is able to develop proteins in the presence or lack of concurrent selection for protein action and improve the expression of a wide range of proteins, along with bacterially derived proteins (MBP), genetically modified antibodies (scFv), and eukaryotic enzymes, as well as proteins with lower stability that have developed via protracted directed evolution. In conclusion, SE-PACE may provide a method for swiftly reversing the negative consequences of large-scale mutations in order to improve the usability of advanced or previously designed proteins (Wang et al., 2018).

The introduction of phage-assisted continuous evolution (PACE) of TEV protease was an early attempt at this, which involves protease generated from approximately 2,500 generations of PACE with 20 non-silent mutations that clears human IL23 at the target peptide bond and suppresses IL23-mediated immune activation when pre-mixed with IL23 in primary mouse splenocyte cultures. The specificity profile of mutation cleavage and an additional protease showed the molecular basis for some changes and supports the creation of custom-made proteases that catalytically change or terminate target proteins for biotechnological and therapeutic applications (Packer et al., 2017). This work establishes not only the ability to change the substrate specificity of a protease at various positions but also on a suitable time scale.

## Conclusion

Lactic acid bacteria not only offer great potential as an established organic product, but as public interest in the importance of probiotic usage in food and health-related aspect rises, researchers of this millennium are increasingly introducing classical therapies into genomic approaches. While the results for recombinant bacteria and their metabolites are relatively mixed, they are anticipated in the near future. The importance of recombinant probiotics has been reported in mouse models, but some few human clinical studies is also encouraging. However, the main problem with the development and usage of probiotics and other well-established organisms lies in the selection of the strains, the appropriate dose, and the lateral transfer of genes from recombinants to other types of bacteria. The use of CRISPR Cas9 selection with LAB recombination tools, PACE, and the continuous introduction of AI-based protocols (machine learning and deep neural network) could be useful in the production of important compounds and the ability to restore health with high efficiency and precision. In summary, *Pediococcus* and other related LAB species remain an effective, safe, and innovative alternative for food, health, and biotechnology applications.
